# Error-enhancing robot therapy to induce motor control improvement in childhood onset primary dystonia

**DOI:** 10.1186/1743-0003-9-46

**Published:** 2012-07-23

**Authors:** Claudia Casellato, Alessandra Pedrocchi, Giovanna Zorzi, Giorgio Rizzi, Giancarlo Ferrigno, Nardo Nardocci

**Affiliations:** 1 Politecnico di Milano, BioengineeringDept., NearLab. Piazza L. Da Vinci 32, 20133, Milan, Italy; 2 Fondazione IRCCS Istituto Neurologico Carlo Besta, Child NeurologyDept., Via Celoria 11, 20133, Milan, Italy

**Keywords:** Force field paradigms, Internal models, Dystonia, Motor adaptation

## Abstract

**Background:**

Robot-generated deviating forces during multijoint reaching movements have been applied to investigate motor control and to tune neuromotor adaptation. Can the application of force to limbs improve motor learning? In this framework, the response to altered dynamic environments of children affected by primary dystonia has never been studied.

**Methods:**

As preliminary pilot study, eleven children with primary dystonia and eleven age-matched healthy control subjects were asked to perform upper limb movements, triangle-reaching (three directions) and circle-writing, using a haptic robot interacting with ad-hoc developed task-specific visual interfaces. Three dynamic conditions were provided, null additive external force (A), constant disturbing force (B) and deactivation of the additive external force again (C)*.* The path length for each trial was computed, from the recorded position data and interaction events.

**Results:**

The results show that the disturbing force affects significantly the movement outcomes in healthy but not in dystonic subjects, already compromised in the reference condition: the external alteration uncalibrates the healthy sensorimotor system, while the dystonic one is already strongly uncalibrated. The lack of systematic compensation for perturbation effects during B condition is reflected into the absence of after-effects in C condition, which would be the evidence that CNS generates a prediction of the perturbing forces using an internal model of the environment.

The most promising finding is that in dystonic population the altered dynamic exposure seems to induce a subsequent improvement, i.e. a beneficial after-effect in terms of optimal path control, compared with the correspondent reference movement outcome.

**Conclusions:**

The short-time error-enhancing training in dystonia could represent an effective approach for motor performance improvement, since the exposure to controlled dynamic alterations induces a refining of the existing but strongly imprecise motor scheme and sensorimotor patterns.

## Background

In the last decades there has been an increasing number of studies investigating how the Central Nervous System (CNS) controls movements, by using robotic technology. Force field paradigms are able to guide internal model formation, i.e. sensorimotor adaptation mechanisms, as form of implicit learning [1]. In particular, multijoint reaching movements have been studied by applying robot-generated deviating forces [2]. Through haptic robots, indeed, the subjects are exposed to extrinsic dynamics (environment), whose effect is added to the effect of the intrinsic one [3-5]. This non-invasive tool allows to accurately observe and quantify the motor response of the CNS to an external dynamic interaction, investigating possible short-term adaptation strategies.

Patton and colleagues [6] exposed stroke patients to robot-generated force fields; they found out that significant improvements occurred only when the training forces magnified the original errors (error-enhancing therapy). A similar approach was employed by Masia and colleagues [7] on cerebral palsy children, with the final goal to identify a tool able to evaluate to which extent their motor learning capability is impaired.

The force field paradigm application on healthy subjects allowed to point out relevant features of motor learning; for instance, differences in motor mechanism tuning between children and adults emerged [8] and a kinematic variability was found as subjects move in different directions while experiencing the same structured force field [9,10], due to the anisotropic features of the arm impedance.

In this framework, the response to altered dynamic environments and the evaluation of motor learning of children affected by primary dystonia have never been investigated. Dystonia is a syndrome characterized by excessive and sustained muscle contraction causing twisting and repetitive movements, abnormal postures, or both [11-13]. New insights suggest that dystonia could also have as origin a lack of reliable sensory feedbacks about motor actions [14], in particular an impairment of sensory integration of afferent inputs, such as proprioceptive signals [15,16]. The ability of dystonic children to deal with the central planning issues associated with the control of arm motion is still an open question [17].

The purpose of the present study is to evaluate whether the application of force to limbs can improve motor learning in children affected by primary dystonia. Indeed, it is well-known that healthy adults can quickly adapt to novel dynamic environments, forming a mapping (an internal model) between limb state and muscle forces; it is less asserted a standard behavior concerning children.

Thus, explorative experimental sessions switching on/off an external force field, on healthy and dystonic children, performing motor tasks with different direction features, allow to outline the adaptation mechanisms and the error signals that drive these processes.

In terms of clinical fall-outs, this investigation explores the possibility of teaching to dystonic patients desired movements using after-effects from adaptation to robot-applied force fields, in particular exploiting the error-amplifying forces, whose potential is justified by the observation that movement error is likely to be a driving signal for adaptation and learning [18,19]. All these insights would suggest whether and how adaptive training could provide an effective supplement to conventional interventions, which can be of limited efficacy, for dystonia. Indeed, the current treatment options include pharmacological and surgical strategies; in children with primary dystonia, which is often generalized, the results may be not satisfactory, not steady in long-term and not effective for all tasks, hence specific rehabilitative trainings are needed for better improvements. Which neurorehabilitative exercises are the most effective for dystonic people is still matter of discussion [20].

## Methods

### Subjects

Eleven children (10 males and 1 female) aged 8 to 18 years with Primary Dystonia (PD) and eleven age-matched healthy control subjects (between 10 and 15 years; 6 male, 5 female), with no neurological or orthopedic impairment, were included in this study. Participants or their parents or guardian gave written informed consent for the study in accordance with the Institutional Review Board of the Istituto Neurologico Carlo Besta. Patients were selected from the Pediatric Movement Disorder Database of the Institute. The inclusion criteria were: diagnosis of primary dystonia, aged more than 6 years and clinical evaluation (severity score) attesting the capability of basically carrying out the required protocols. Nine patients had a *DYT1* negative primary dystonia, two patients had a *DYT1+* primary dystonia [21]. Seven patients were chronically stimulated with bilateral DBS (Deep Brain Stimulation) in globus pallidus. At time of testing the stimulation parameters were as follows; amplitude: 2–4.5 V; pulse width: 90–330 μsec; frequency: 130–185 Hz. Two patients were under pharmacological treatment (trihexyphenidyl) and two were medication off.

Concomitant to the recording session, all patients were administered the Burke-Fahn-Marsden Dystonia Rating Scale by two experts in pediatric movement disorders (N.N. and G.Z.). Detailed information of the 11 patients are reported in Table 1.

**Table 1:** General and clinical information of the 11 patients

**Subject**	**Gender**	**Age**	**Dystonia**	**Therapy**	**Severity**	**UL**_**r**_	**UL**_**l**_
1	m	18	DYT1 [−]	DBS	50	12	16
2	m	8	DYT1 [−]	none	15	0	3
3	m	12	DYT1 [−]	trihex	34	4	9
4	m	17	DYT1 [−]	DBS	26	4	6
5	m	18	DYT1 [−]	DBS	50	4	4
6	m	15	DYT1 [+]	DBS	20	2	0
7	f	12	DYT1 [−]	DBS	11	2	1
8	m	14	DYT1 [−]	none	18	4	0
9	m	16	DYT1 [−]	DBS	20	6	4
10	m	9	DYT1 [+]	tryhex	46	16	6
11	m	18	DYT1 [−]	DBS	14	4	2

DBS: Deep Brain Stimulation. The Burke-Fahn-Marsden Dystonia Rating Scale (BFMDRS) scores are indicated as Severity and Upper Limb severity (UL for right and left sides). Range of Severity: 0–120; range of UL severity: 0–16.

### Experimental set-up

**Figure 1:**
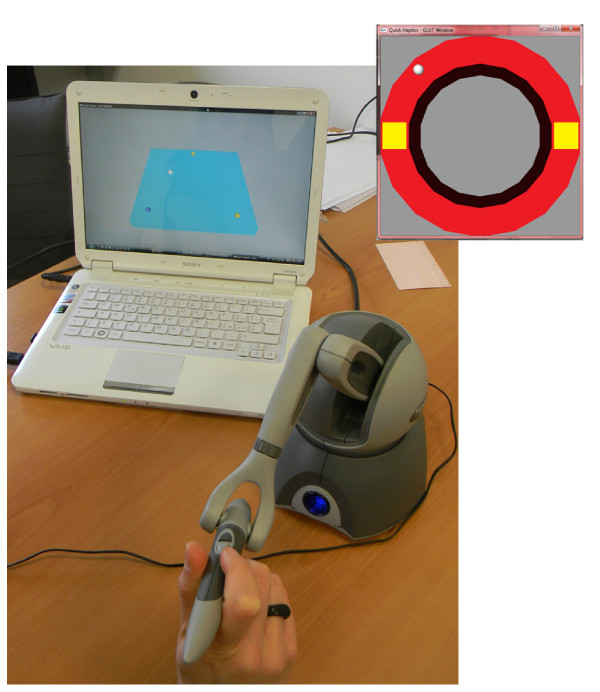
**Haptic-visual set-up.** Triangle-reaching task performed by a subject, using the PHANToM OMNI (SensAble™). At the right top, the circle-writing interface.

The set-up (Figure 1) was focused on a visual-haptic interface based on the device PHANToM OMNI (SensAble™). It is an electromechanical device for kinesthetic active force feedback on the axes x, y and z, from the measurement of instantaneous position and velocity (optical encoders representing proprioceptive sensors). From the angles of each actuated joint, the end-effector Cartesian coordinates are computed, by direct kinematic method. Then, the relative position between the user and the virtual object is used to define the interactions and thus the reaction force; by multiplying this force to the transpose Jacobian matrix, the joint torques are calculated. The kinesthetic stimulus in the PHANToM OMNI pencil is so recreated. The most important technical specifications of the PHANToM OMNI haptic device correspond to a nominal resolution of 0.055 mm offset in its workspace and an end-effector nominal force of 3.3 N. The communication interface is IEEE-1394 Fire Wire port and allows real-time programming in Visual C++. The developed control algorithm updates the virtual environment and records the pointer 3D position at 50 Hz.

Three experimental conditions were tested:

A: null additive external force (reference)

B: constant disturbing force along x-axis, i.e horizontal (intensity = 1.3 N)

C: deactivation of the additive external force again

The force field intensity was chosen from preliminary acquisitions, taking into account the number of falling from the task surface, the execution time and subjects’ fatigue perception.

### Tasks

For our set-up, we developed graphical interfaces that define the motor tasks: triangle-reaching and circle-writing tasks. It allows to explore how the motor adaptation generalizes between different-direction tasks (straight reaching and circles drawing) that share the same workspace [22]. The user moving into the virtual environment is depicted as a white sphere. The subjects sat on a chair, at a comfortable distance from the device and screen.

The reaching interface was made up of a virtual table, with haptic features, inclined at a 45° angle, on which three fixed spheres were placed, as triangle vertexes (Figure 1). For each experimental condition, the subject was asked to draw the triangle continuing to touch the table surface. To complete each of the three tracts the user had to reach the vertex; such event was visualized by the change of sphere color. The side lengths of the isosceles triangle were 100, 100, 130 mm.

The circle-writing interface was made up of a circular-ring virtual surface, with haptic proprieties. The subject was asked to complete the circle drawing, keeping on to touch the guide surface (Figure 1). Two square elements were placed along the guide, to provide, through a color code, a check about the surface touching and about the trial completion. The external circumference of the ring was 210 mm length and the internal one 160 mm.

For each experimental condition, 15 consecutive trials were asked.

The acquisitions were preceded by a familiarization phase, made up of 8–10 trials for each task, to allow an understanding of the device working. No constraints about the movement speed were indicated.

**Figure 2:**
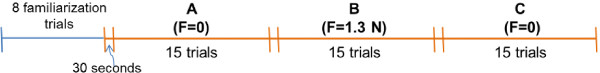
**Timing scheme of the protocol.** Sequence of repetitions for each of the 3 conditions (**A**, **B** and **C**).

Figure 2 depicts the protocols timing.

### Data analysis

Data analyses were performed by using custom software developed in Matlab (Mathworks Inc.) and Statistica (StatSoft).

For both tasks, each single trial (each tract of reaching and each circle) was identified through the recorded events (touchable elements, i.e. vertex spheres and squares).

For reaching, for each direction/tract, the path length was computed from the recorded position data. It represents a quantification of “deviation error” compared to the straight trajectory.

For circle-writing, for each trial, the path length was computed too.

#### *Statistical analyses*

For both protocols, the following non-parametric tests, more conservative than the parametric ones, were performed:

Mann–Whitney test, between-groups and within-condition. That is, it was performed for each condition separately, comparing dystonic vs. healthy, in order to detect any distinguishable features of the dystonic population.

Wilcoxon test, within-group and between-conditions (paired samples, i.e. each subject). That is, it was performed for healthy and dystonic groups separately, comparing the conditions (A vs. B vs. C), in order to detect any effect of force field changes on each subject’s movement behavior.

Wilcoxon test, within-group and within-condition (paired samples, i.e. each subject). That is, it was performed for healthy and dystonic groups separately and for each condition separately, comparing the first and the last trials (first 4 trials vs. last 4 trials), in order to detect any adaptation trend, along task repetitions in the same condition.

## Results

**Figure 3:**
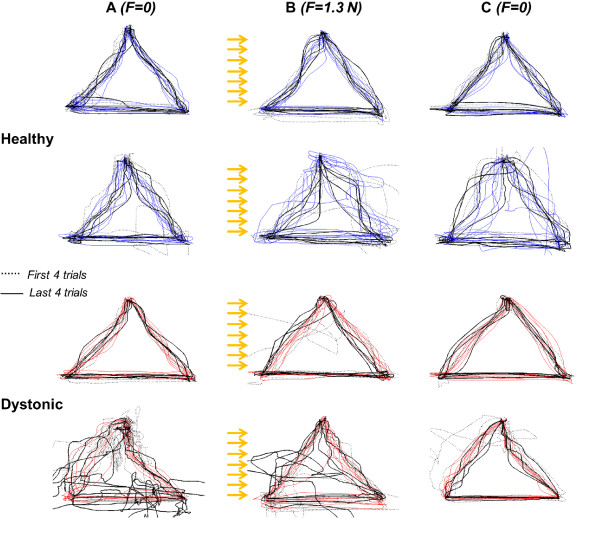
**Triangle-reaching task.** Exemplificative trajectories of two healthy and two dystonic subjects (the most uniform and least uniform among both dystonic and healthy subjects), in the 3 conditions (**A**, **B** and **C**). The first four and the last four trials are indicated in black dashed and thick lines, respectively, while the intermediate ones in blue for healthy and in red for dystonic. The yellow arrows represent the disturbing force along x-axis, i.e. horizontal.

All the subjects were able to perform the assigned tasks in the different experimental conditions. Figure 3 shows the reaching trajectories in the three dynamic conditions for representative subjects from healthy and PD groups, respectively. In all conditions, the unimpaired group control was more effective, leading to straighter trajectories, than the dystonic one.

**Figure 4:**
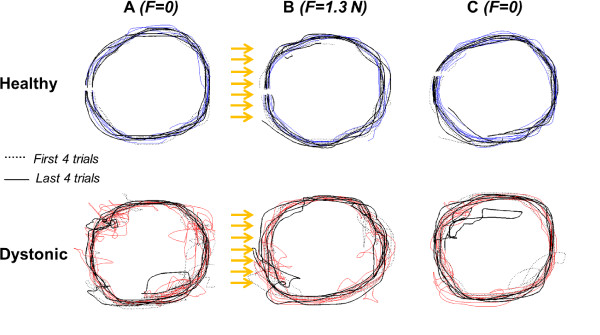
**Circle-writing task.** Exemplificative trajectories of one healthy and one dystonic subject, in the 3 conditions (**A**, **B** and **C**). The first four and the last four trials are indicated in black dashed and thicklines, respectively, while the intermediate ones in blue for healthy and in red for dystonic. The yellow arrows represent the disturbing force along x-axis, i.e. horizontal. Note: for the few trials where the subjects lost the leaning on the virtual surface, the data portion where the user end-effector was not touching the surface was cut (event detection); and such trial was reconstructed and included into the analysis if the re-started path passed back for the falling point.

Figure 4 shows the trajectories for the circular path for representative subjects. Control subjects had better performances in terms of smoothness of trajectories than PD group.

### Triangle-reaching task

**Figure 5:**
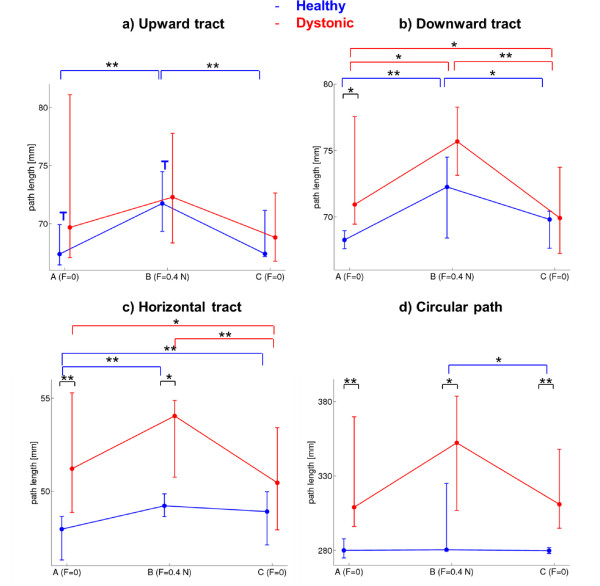
**Path lengths.** Path length of reaching (three tracts: panels **a**, **b** and **c**) and of circle-writing (panel **d**) for the three force field conditions (A-B-C on x-axes). In blue: median and 75-*th* and 25-*th* percentiles interval among healthy subjects. In red: median and 75-*th* and 25-*th* percentiles interval among dystonic subjects. There are indicated the significant differences between conditions for each group (* for p < 0.05 and ** for p < 0.01, by Wilcoxon test), the significant differences between groups for each condition (* for p < 0.05 and ** for p < 0.01, by Mann–Whitney test) and the significant trends of adaptation for each group and for each condition (“T”, when p < 0.05 by Wilcoxon test between first 4 trials and last 4 trials).

For healthy subjects (blue elements in Figure 5), the disturbance affected significantly the optimal trajectory control. Indeed, for all the three reaching tracts (Figure 5a-b-c), the deviation of trajectories from the straight line in B condition was significantly higher than in A condition (A vs B. Upward: Z = 2.93, p < 0.01; downward: Z = 2.67, p < 0.01;horizontal: Z = 2.58, p < 0.01). Concerning B condition, only in the upward tract (Figure 5a), a significant adaptation Trend was found (Z = 2.13, p < 0.05, indicated by “T” on the figure), quantified as a difference between first and last trials. A significant adaptation was found also in A condition of this upward tract for healthy subjects; despite of the familiarization phase, this reaching direction was characterized by a progressive achievement of a more and more straight trajectory. Thus, since a significant tendency to compensate for the force, that is to carry out a straighter trajectory, was turned out only in the upward tract, it can be suggested that the adaptation process is direction-dependent in healthy children; indeed, with the same number of trial repetitions, it was not equal for the three reaching directions.

Consistently with a general lack of within-condition adaptation, the C condition did not show the so-called after-effects [2,5], i.e. its kinematic was not characterized by a higher curvature than the reference condition, neither an adaptation trend emerged. This condition is characterized by an immediate complete recovery of the path control as in the reference environment.

The dystonic behavior (red elements in Figure 5) was significantly different from the healthy one. In general, the path control was strongly compromised (high deviation) in all conditions, and the disturbance did not affect systematically the motor control as in healthy population. Indeed, in the upward and horizontal tracts (Figure 5a-c), no significant difference between A and B conditions was revealed. Only in the downward tract (Figure 5b) this disturbance impact was relevant (Z = 2.04, p < 0.05).

Moreover, no within-condition adaptation, always quantified by significant differences between early and late phases, was present in any experimental condition.

What emerged is that the disturbing force field phase seems to induce a subsequent improvement of path control, shown in C condition. Indeed, in all the three reaching directions, the median path length of the dystonic population in C condition is lower not only than in B but also than in A conditions. The downward and horizontal tracts (Figure 5b-c) show statistically a straighter trajectory in C than in A conditions (Z = 2.13, p < 0.05; Z = 2.04, p < 0.05, respectively).

The between-groups comparison in each condition confirms the observation that the disturbance phase induces a refining of the reference internal model in dystonic children. Indeed, the dystonic and the healthy populations are closer in C condition than in A and B ones. In downward and horizontal tracts, the significant difference between groups evident in A condition (Z = 2.4, p < 0.05; Z = 2.86, p < 0.01, respectively) disappeared in C condition.

In terms of duration, in each condition, the 15 consecutive trials were completed in: Healthy, A: 85 (28), B: 87 (28), C: 87 (28) seconds. Dystonic, A: 155 (99), B: 158 (98), C: 157 (96) seconds.

### Circle-writing task

For the more complex task of circular path tracking, the disturbance did not have a systematic effect either on healthy or on dystonic subjects. As depicted in Figure 5d, the only between-conditions comparison resulting into a significant difference was between B and C conditions for healthy children (Z = 2.4, p < 0.05), who basically showed an inter-subjects variability increase during the altered force field movements and an inter-subjects variability decrease when they came back to null field, compared to reference behavior. The altering force impact continuously changes on a curvilinear trajectory, leading to phases of the trial where the path tracking is facilitated and where it is hampered.

The most evident finding from the circle-writing protocol concerns the comparison between the two groups. In all conditions, the two median behaviors were significantly different (dystonic vs healthy in A: Z = 2.99, p < 0.01; in B: Z = 2.27, p < 0.05; in C: Z = 2.92, p < 0.01): this path shape task leads to a bigger distinction between the dystonic behavior and the healthy one than the reaching task, regardless the environmental force field.

In terms of duration, in each condition, the 15 consecutive trials were completed in: Healthy, A: 84 (25), B: 76 (26), C: 61 (15) seconds. Dystonic, A: 138 (55), B: 115 (54), C: 98 (46) seconds.

## Discussion

This paper explores the features of motor adaptation in children with primary dystonia during the execution of multijoint movements that are disturbed by a force field, comparing their behavior with an aged-matched control group. Both groups completed the required tasks. However, there are clear evidences of different performances between impaired and unimpaired children.

Our findings from healthy subjects show that the disturbing force affects significantly the movement outcomes. Moreover, while the dynamic alteration is acting, a complete compensation is not achieved, i.e. the trajectory does not return to be as straight as in the reference condition. Fifteen repetitions, not consecutive but inserted into a direction-change sequence for the reaching task, do not allow to complete the updating of the internal model of body/environment which drives the feedforward control mechanism. Only in upward reaching a tendency to carry out compensatory strategies, canceling the constant perturbation effect on the trajectory, emerged.

The absence of systematic adaptation during the divergent force field in healthy children could be better investigated in order to understand how much it is ascribable to the defined timing of the protocol phases or to the young age, since it is known that, to optimize motor learning, children may require longer periods of practice than adults [8,23].

Alternatively, it is possible that the motor behavior is not recovered as a matching with initial reference performance (A condition). According to the theory proposed by Izawa and colleagues [24], the motor control in a novel environment is not a process of perturbation cancellation, rather, the process resembles reoptimization. Thus, it would support the hypothesis that the control of action proceeds via two related pathways: on the one hand, adaptation produces a more accurate estimate of the sensory consequences of the motor commands (i.e. learning an accurate forward model), and on the other hand, the brain searches for a better movement plan which minimizes an implicit motor cost and maximizes rewards (i.e. finding an optimum controller).

Anyway, the lack of adaptation in B condition is consistent with the lack of after-effects in C condition, when the disturbance is re-deleted; indeed, the motor control when the dynamic alteration is not acting anymore is perfectly comparable with the reference one. Also Burdet and colleagues [25] found for healthy subjects performing reaching that, when the force field was removed, the trajectories were even straighter than in the reference condition: after-effects were absent following adaptation to a destabilizing force field that amplified trajectory errors. It was associated to the CNS skill of tuning impedance, in order to achieve stability.

Thus, for healthy group, the disturbance uncalibrates the sensorimotor system. It is known that internal models, i.e. body/environment representations, are efficient for motor control only if they produce unbiased predictions of body states; it requires that the level of noise in the system is sufficiently low and mainly that the sensorimotor system is well calibrated [26,27].

In the light of what we observed about the healthy controls, the dystonic subjects show a less efficient internal model, regardless the external disturbance. In fact, in the reference condition without any disturbing factors, they carry out a less precise, more variable and less reliable path control than the healthy ones. Since their sensorimotor system is already not-well calibrated, the disturbance does not systematically affect their motor control. It has been shown that dystonia is characterized by abnormal sensory integration, i.e. by an incomplete processing of the incoming signals, resulting in distorted information and thus in abnormal motor outputs [15].

However, it is particular intriguing how the altered dynamic exposure (B condition) would induce a subsequent improvement (C condition), in terms of optimal path control, in dystonic population, despite a lack of clear adaptation during the exposure to the constant disturbance.

Rossetti and colleagues [27] speculated about the mechanisms that might allow a stroke subject to decrease directional errors with force field paradigm based on distorting interventions, that cannot decrease by simple practice alone: sensory feedback systems may need to detect a stimulus with a magnitude that is large enough to trigger the recovery process [28-30]. Many of the substrates related to robot-mediated motor adaptation overlap with brain areas related to motor recovery after a CNS injury such as stroke [31,32]. Masia and colleagues [7], interpreting such mechanisms in children with cerebral palsy, proposed another explanation: the nervous system is trying to use motor pathways that are no longer intact, and the learning is a way to trick the nervous system into a new and non-intuitive pathway that it would otherwise not ever consider; however, such ideas would need movement-correlated brain imaging studies to be validated [33,34]. Speculating on our findings in the same framework, we transfer these considerations on dystonia; the external sensory element could induce a “motor control improvement” by enhancing the sensorimotor system calibration. In other words, a very short-term environment alteration is probably not able to establish new sensory engrams for the new dynamic environment (i.e. specific learned and memorized motor patterns stored in both sensory and motor portions of the brain), but it is able to refine the sub-optimal standard sensorimotor patterns for a specific task. The suggestion is that the disturbance could not induce single trials evolution, but a global effect triggered by the just-experienced dynamic alteration. The force-field paradigm induces adaptation in a relatively short timescale, on the order of tens of movements, which makes it possible to perform motor-learning experiments quickly [35].

The uncalibrated dystonic sensorimotor system seems to have improvement margins: the force alterations would induce a more effective recruitment of cortico-cortical connections linking the ipsilateral motor and somatosensory cortical areas. The short-time error-enhancing therapy in dystonia could represent a training to refine the existing but strongly imprecise motor scheme.

As for healthy, the behaviour in C condition for dystonic children does not show after-effects. Differently from the healthy CNS which can control impedance to enhance the robusteness to external perturbations or to biased sensorimotor transformations [36], dystonic subjects could not be able to control the full impedance, which would mean to minimize the metabolic cost [25]; they likely act only on stiffness control, which is realized by prolonging the movement duration and/or co-contracting muscular activity, thus causing bradykinesia and increased motor output variability [12,37]. Trial-to-trial variability indeed arises from neural sources [38,39] and is larger during childhood [40] or due to a brain damage.

These underlying mechanisms, both in healthy and in dystonic subjects, should be better investigated also in relation to the directionality of motor learning, about which here some cues emerged from reaching tasks. For more robust conclusions, different force field paradigms (e.g. different directions and intensities), even subject-specific, should be tested and wider populations with systematic features, possibly untreated patients, should be recruited.

The main limitation of this work is the quite small number of trials; this choice in the protocol definition arose by the need to not introduce fatigue and demotivation which could confuse the comparisons between the consecutive conditions. Moreover, most of the dystonic children were under treatment (medical and/or deep brain stimulation) and they displayed a great variability in the severity of dystonia, even if all affected homogeneously by primary dystonia. Another major issue to be addressed in details in the next related studies is the persistence of such potential beneficial effects after a sequence of training sessions exposing the patient to dynamic alterations and waiting for a washout period.

The basic scientific knowledge gained with the robotic force-field paradigm most likely will lead to practical enhancements in motor learning issues. A thorough understanding of the error signals that drive adaptation may allow them to be amplified or filtered, thus accelerating learning.

In conclusion, this work highlights encouraging evidence that haptic training could provide an effective supplement to conventional therapy in dystonia. Thus, the neural processes associated with implicit motor adaptation may reshape sensorimotor mappings altered by dystonia that cannot be tuned simply by practicing movement.

## Competing interests

The authors have no competing interests in relation to this study.

## Authors’ contributions

CC participated to conception, organization and execution of the research project, set-up development, data collection, design and execution of statistical analysis, manuscript writing. AP participated to organization of research project, study design and manuscript writing. GR participated to set-up development, data collection and analysis, and manuscript writing. GZ participated to organization and execution of the research project, manuscript writing. GF participated to study design and realization. NN participated to conception and organization of research project, review of clinical and pathophysiological aspects, critical review of the manuscript. All authors read and approved the final manuscript.
